# Supplementary material for: MHC-mismatched synovial mesenchymal stem cell injections delay knee osteoarthritis progression through hepatocyte growth factor secretion in rats
Source: PLoS One. 2025 Oct 3;20(10):e0333117. doi: 10.1371/journal.pone.0333117 (PMC12494247; doi:10.1371/journal.pone.0333117)
Supplement: S1 File — (DOCX) [file pone.0333117.s001.docx]

S1 File. The ARRIVE guidelines 2.0: author checklist.
