# Supplementary material for: MHC-mismatched synovial mesenchymal stem cell injections delay knee osteoarthritis progression through hepatocyte growth factor secretion in rats
Source: PLoS One. 2025 Oct 3;20(10):e0333117. doi: 10.1371/journal.pone.0333117 (PMC12494247; doi:10.1371/journal.pone.0333117)
Supplement: Attachment — Submitted filename: Kitahashi Allo Plos Rebuttal 250816 1807.docx [file pone.0333117.s003.docx]

August 16, 2025

Dr. Xindie Zhou

Academic Editor

PLOS ONE

Dear Dr. Zhou,

Thank you for your comments from July 4, 2025. Based on these valuable comments, we have rewritten our manuscript, including the title. The new title is "MHC-mismatched synovial mesenchymal stem cell injections delay knee osteoarthritis progression through hepatocyte growth factor secretion in rats."

We believe that the revised paper is greatly improved. The following are our responses to the specific points made by your reviewers.

We would very much appreciate having our revised paper considered for publication in Scientific Reports.

Sincerely yours,

Ichiro Sekiya, MD, PhD

Director, Center for Stem Cell and Regenerative Medicine

Professor, Department of Applied Regenerative Medicine

Institute of Science Tokyo (IST)

1-5-45 Yushima, Bunkyo-ku, Tokyo, 113-8510, Japan

Tel: +81-3-5803-4017

Fax: +81-3-5803-0192

Email: sekiya.arm@tmd.ac.jp

Review Comments to the Author

Reviewer #1:

The study asks whether major histocompatibility mismatch in synovial-derived MSCs will block the chondroprotective effect previously shown by the same group in a different rat OA induction model.

The study also asks whether the synovial-derived MSC’s ability to express HGF is necessary for their therapeutic effect.

The major findings of the study are that synovial derived MSCs appear to still have a chondroprotective effect despite MHC mismatch; and that this effect was not observed if synovial derived MSCs with siRNA induced HGF knockdown were used.

Overall comments:

The strengths of the study are the well-controlled designs, reproducibility obtained, and careful analyses.

The weaknesses of the study are the lack of translational relevance of the ACLT model as a clinical model of human OA induction in which damage is much more common in the femoral condyle;

We acknowledge that the ACLT model does not fully replicate the spatial distribution of cartilage damage typically observed in human OA, where the femoral condyle is often more severely affected. However, the ACLT model remains a widely accepted and standardized model for post-traumatic osteoarthritis and provides consistent induction of joint instability and inflammation. Importantly, our aim in conducting this study was not to replicate the entire clinical presentation of OA, but to evaluate the relative therapeutic contribution of HGF within synovial MSCs under immunologically challenging conditions using MHC-mismatched transplantation. In this context, the ACLT model offers a reproducible and appropriate platform for assessing therapeutic differences in MSC-based interventions. We have added the following paragraph to the Discussion:

“Several limitations of this study should be noted. One was that we used the anterior cruciate ligament transection (ACLT) model, which, although widely adopted for post-traumatic osteoarthritis research, may not fully recapitulate the complex pathology of human OA. While this model allows for reproducibility and controlled OA induction, its translational relevance remains limited [26].”

the lack of translational relevance of the MSC injection regimen as a clinical injection regimen where repeated injections would not be feasible;

We acknowledge the reviewer's comment regarding the translational limitations of the repeated MSC injection regimen used in this study. While multiple intra-articular injections may not be routinely feasible in clinical settings due to concerns about patient burden and cost, they are commonly employed in preclinical studies to maximize the opportunity for detecting therapeutic effects and to understand dose-response dynamics under controlled conditions.

Our goal in using repeated injections was to ensure sufficient exposure of transplanted MSCs within an immunologically challenging environment (MHC-mismatched model) and to achieve a robust assessment of the contribution of HGF to therapeutic efficacy. This design helped reduce variability and clarify the biological role of HGF in the context of allogeneic MSC therapy. Nonetheless, we agree that single-dose or limited-injection regimens would more closely reflect clinical practice, and we have noted this as a limitation in the revised Discussion section, as follows:

“Another limitation was that we administered synovial MSCs via intra-articular injection once weekly for 7 weeks, which differs from typical clinical protocols where such frequent dosing may not be feasible [4]. Future studies should explore more clinically relevant dosing regimens.”

and lack of consideration of other signals affected by HGF knockdown in the MSCs that may be impairing their activity versus the author’s inference that the chondroprotective activity of the cells is due exclusively to HGF knockdown.

We thank the reviewer for this insightful comment. We fully agree that HGF knockdown may influence more than just HGF secretion and may also affect other signaling pathways within MSCs, potentially contributing to the observed reduction in therapeutic efficacy. Our study was designed as an observational investigation to explore the role of HGF in MSC-mediated chondroprotection under MHC-mismatched conditions rather than as a study that would definitively establish mechanistic causality.

To avoid overinterpretation, we have revised the relevant statements in the Discussion to clarify that while our findings suggest a contribution of HGF to the therapeutic effects, they do not exclude the possibility that additional molecular changes associated with HGF knockdown may also play a role. We have also added this point as a limitation of the study. Based on these reviewer comments, we have substantially revised the Limitations section as follows:

“Our mechanistic analysis focused specifically on HGF function through siRNA knockdown. While this approach demonstrated the contribution of HGF to MSC efficacy, we did not conduct a comprehensive evaluation of whether *Hgf* knockdown affected other chondroprotective factors, such as PRG4 or BMP2/6 [3], nor did we perform rescue experiments to isolate the specific contribution made by HGF. Future studies incorporating secretome analysis and rescue experiments would provide more definitive mechanistic insights.”

Specific comments:

Describe size of needle used in MSC injections.

We have added the needle size used for MSC injections as follows:

“Under isoflurane anesthesia, a 28-gauge needle was used for intra-articular injection of 50 μL of the following assigned treatments into the knee joints of the animals in each group:”

Describe if the MHC mismatched cell injections led to any immune response in the animal – eg, presence of lymphocytes in the synovium should be examined.

We thank the reviewer for this important comment regarding the assessment of immune responses following MHC-mismatched MSC administration. We agree that evaluating immune cell infiltration, particularly lymphocytes in the synovium, would provide valuable insights into the immunological consequences of allogeneic MSC transplantation.

In response to this comment, we have added a section titled "Knee findings after cell administration" to describe our observations regarding potential immune responses. As detailed in this new section, we assessed immune responses by examining knee swelling and joint tissue hypertrophy during postmortem examination following synovial MSC administration. However, we observed joint swelling and tissue thickening uniformly across all groups, with no specificity to the cell-administered groups, suggesting that these changes were primarily related to the ACLT-induced osteoarthritis rather than to immune rejection of the transplanted cells.

We have added the following paragraph to the Results:
“**Knee findings after cell administration**

The induction of immune responses following the administration of MHC-mismatched cells was assessed by examining knee swelling and joint tissue hypertrophy during postmortem examination after synovial MSC administration. However, joint swelling and tissue thickening were observed uniformly across all groups and were not specific to the cell-administered group.”

The authors have previously identified Prg4 and BMP2,6 as additional potentially pro-chondrogenic factors expressed by synovial MSCs. Were the levels of Prg4 and BMP2,6 mRNA and protein also decreased in the cells by HGF knockdown? Without this information, it is an overstatement to attribute the chondroprotective.

We thank the reviewer for this important and insightful comment. We fully agree that the potential involvement of other pro-chondrogenic factors, such as Prg4 and BMP2/6, in mediating the chondroprotective effects of synovial MSCs warrants further investigation. However, due to time constraints—specifically, the limited five-week period allocated for the revision—performing additional experiments involving mRNA and protein analysis of these factors was not feasible within the current study timeline. We plan to address this important mechanistic question in a follow-up study, and we have added the following statement acknowledging this limitation to the Discussion section:

**“**Our mechanistic analysis focused specifically on HGF function through siRNA knockdown. While this approach demonstrated the contribution of HGF to MSC efficacy, we did not conduct a comprehensive evaluation of whether *Hgf* knockdown affected other chondroprotective factors, such as PRG4 or BMP2/6 [3], nor did we perform rescue experiments to isolate the specific contribution made by HGF. Future studies incorporating secretome analysis and rescue experiments would provide more definitive mechanistic insights.”

Reviewer #2:

This manuscript presents a study aimed at determining the implication of hepatocyte growth factor (HGF) in the therapeutic effects of synovial derived mesenchymal stem cells (MSCs) in a rat osteoarthritis (OA) model. To do this, both in vitro and in vivo studies have been conducted. First, HGF expression is silenced, and it is demonstrated that this silencing has a negative effect for the chondrogenic differentiation of synovial MSCs in vitro. Second, both HGF-silenced and control MSCs are administered into rat OA knees (as well as no-cells control), and the effects of these administrations is measured by macroscopic and histological findings in cartilage. Of note, the in vivo study is designed for the MSC administration to be MHC-mismatched, using two different rat strains as donors (ACI) and recipients (Lewis).

Overall, the manuscript is well written, the goals are clear (but could be more precisely defined), the study design and methods are appropriate (they could be complemented, though), and the results are generally clearly presented. Limitations are discussed and conclusions are based on the results. In general, this is an interesting and formally adequate manuscript, however some points need attention prior to further considering it for publication.

Please see below main general comments, and later more specific comments pointed out along the manuscript. I hope these points can help authors making the most of their manuscript, but if they disagree with any comment/suggestion, please kindly explain why.

General comments:

- I strongly recommend putting more emphasis into the novelty of the study. The introduction is very brief, and, in my opinion, it does not provide enough context to understand the relevance of the present work. Previous studies have already assessed the role of HGF in MSC mechanisms in vitro and in vivo, and some of them have also assessed knockdown cells in different disease models, so the authors should answer very clearly in the introduction: what is the novelty of our study? What does our study adds to the current knowledge?

We sincerely thank the reviewer for this valuable feedback regarding the novelty and context of our study. We completely agree that the previous introduction was insufficient in establishing the unique contribution of our work and its relevance to the current body of knowledge.

In response to this comment, we have substantially expanded and restructured the introduction to better contextualize our study within the existing literature and to more clearly articulate its novelty. The revised introduction now provides a comprehensive background on osteoarthritis pathophysiology, the advantages and challenges of allogeneic MSC therapy, and the specific knowledge gaps that our study addresses.

Specifically, we have clarified that while previous studies have examined HGF's role in MSC mechanisms, our work uniquely focuses on the functional importance of endogenously secreted HGF under fully MHC-mismatched allogeneic conditions—a clinically relevant scenario that remains poorly understood. Unlike previous studies that primarily investigated HGF overexpression or exogenous HGF administration, our approach uses loss-of-function analysis through siRNA knockdown to determine whether endogenous HGF secretion is essential for MSC therapeutic efficacy in an immunologically challenging environment.

Furthermore, we have emphasized that our study addresses critical translational questions, namely, whether the therapeutic benefits of synovial MSCs can be maintained even when donor-recipient immune compatibility is compromised and what role HGF plays in this context. Knowing the answers to these questions is essential for advancing allogeneic MSC therapy toward clinical application, where perfect MHC matching is often impractical.

We have rewritten the introduction as follows:

**“Introduction**

Osteoarthritis (OA) is a degenerative joint disease characterized by cartilage degradation, synovial inflammation, and structural changes in the subchondral bone that ultimately lead to joint dysfunction and chronic pain [1]. OA progression is driven by multiple factors, including cartilage matrix breakdown, chondrocyte apoptosis, and elevated levels of inflammatory cytokines [2]. Due to this multifactorial pathophysiology, therapeutic approaches that can target several pathways simultaneously—such as cell-based therapies—are attracting growing interest.

Synovial mesenchymal stem cells (MSCs) have shown promising chondroprotective effects when administered intra-articularly, as demonstrated in both preclinical [3] and clinical studies [4]. We previously reported that intra-articular injection of autologous synovial MSCs can suppress OA progression in humans [4]. However, autologous transplantation presents several challenges, including donor site morbidity, extended culture periods, and high manufacturing costs. These limitations have prompted increasing interest in allogeneic MSC therapy, which offers advantages in terms of availability, standardization, and scalability.

A key concern arising during allogeneic MSC therapy is immunological incompatibility between the donor and recipient, particularly regarding major histocompatibility complex (MHC) antigens. Previous studies using rat meniscectomy models have shown that meniscal regeneration is poorer following transplantation of fully MHC-mismatched MSCs than following transplantation of syngeneic or partially matched MSCs [5, 6]. This observation suggests that immune rejection can negatively impact therapeutic outcomes; however, the efficacy of using fully mismatched MSCs in OA models remains poorly understood.

Given the limited understanding of the underlying mechanisms controlling the therapeutic responses observed following the transplantation of MHC-mismatched MSCs in OA, clarifying the roles of paracrine factors secreted by MSCs becomes essential. Among the various paracrine factors, hepatocyte growth factor (HGF) is of particular interest due to its well-documented anti-inflammatory, antifibrotic, and tissue-regenerative properties [7]. HGF stimulates chondrocyte proliferation, enhances matrix production, and suppresses catabolic cytokines, and the benefits of the administration of exogenous HGF or HGF-overexpressing MSCs have been reported [8, 9]. Nevertheless, the functional importance of endogenously secreted HGF—especially under MHC-mismatched allogeneic conditions—remains unclear. However, the possibility remains that HGF may serve as a critical mediator of MSC therapeutic efficacy, even in immunologically challenging environments.

The aim of the present study was to clarify the functional role of HGF in establishing the chondroprotective effects of synovial MSCs under fully MHC-mismatched allogeneic conditions. We hypothesized that HGF serves as a key mediator of MSC efficacy, even under immunologically challenging conditions. To test this hypothesis, we employed a rat OA model using ACI rats (donors) and Lewis rats (recipients), which differ markedly in MHC class I and II antigens and are known to elicit robust immune responses [5]. Synovial MSCs were transfected with Hgf siRNA and injected intra-articularly, and the effects of HGF suppression on both in vivo chondroprotective efficacy and in vitro chondrogenic capacity were evaluated. Importantly, our study specifically focuses on OA cartilage rather than meniscal tissue, and employs HGF knockdown to mechanistically dissect the role of this specific paracrine factor under stringent immunological conditions—an approach not previously undertaken in fully MHC-mismatched settings.”

- To assess the effect of MHC-mismatched administration is not an actual goal of the study, but part of its design. While I fully agree that allogeneic MHC-mismatched administration is the most realistic scenario for clinical use, and thus it is important to assess MSCs in this scenario, this study does not really provide information about the effect of MHC-mismatching because no MHC-matched (or syngeneic) control is used. Thus, I recommend placing the focus towards accounting for this factor by selecting a mismatched scenario, but not towards unveiling the effect of such scenario. This should be reflected along the whole manuscript but especially in the goals (end of introduction), study design (beginning of the methodology) and conclusions.

In response to this comment, we have rewritten the objective in the Introduction, as mentioned above. We have also modified the study design at the (beginning of the methodology section) as follows:

“To establish MHC-mismatched synovial MSCs, 30 four-week-old male ACI rats (ACI/N Slc), weighing 50–70 g, were purchased from Japan SLC, Inc. (Hamamatsu, Japan). Fifteen of these rats were used for pooled synovial MSC preparation for transplantation, and the remaining 15 were used to confirm the tissue harvesting protocol.

To evaluate regenerative efficacy and underlying molecular mechanisms, 34 female Lewis rats (LEW/CrlCrlj), aged 9–10 weeks and weighing 150–170 g, were obtained from Jackson Laboratory Japan, Inc. (Yokohama, Japan). Four of these animals were procured as potential replacements for contingency use.”

We have also changed the initial summary in the Discussion as follows:

“This study evaluated the role of hepatocyte growth factor (HGF) in the chondroprotective effects of synovial mesenchymal stem cells (MSCs) under fully MHC-mismatched allogeneic conditions. Using a rigorous ACI-to-Lewis rat OA model, we demonstrated that synovial MSCs retained their therapeutic efficacy despite the complete mismatch in MHC class I and II antigens. Importantly, siRNA-mediated knockdown of Hgf impaired both in vitro chondrogenic differentiation and in vivo cartilage protection. These findings suggest that the beneficial effects of synovial MSCs in this immunologically challenging setting are at least partially dependent on HGF, which appears to support cartilage matrix production and maintain MSC functionality, even in the presence of strong allogeneic immune responses.”

We have also modified the conclusions as follows:

**“Conclusions**

We have established that synovial MSCs can effectively function as therapeutic agents for OA treatment across major histocompatibility barriers, with HGF identified as an essential molecular mediator of their chondroprotective effects. The successful therapeutic outcomes observed in the fully MHC-mismatched ACI-to-Lewis rat model demonstrate the clinical potential of allogeneic MSC therapy, especially for expanding donor availability and treatment accessibility. Our mechanistic analysis through HGF knockdown definitively confirmed that HGF secretion is indispensable for MSC-mediated cartilage protection, thereby establishing this growth factor as a key therapeutic target. Our results support the clinical translation of allogeneic synovial MSC therapy as an OA treatment and suggest that HGF pathway modulation could enhance therapeutic outcomes in regenerative medicine approaches for cartilage disorders.”

- Evaluation of the in vivo study outcome mostly relies on histopathological assessment, and only cartilage is assessed. It could have helped elucidating HGF-mediated mechanisms to include more biochemical parameters about cartilage composition, and also assessing synovium as a key target of MSC therapy to modulate inflammation in OA. I consider that this should at least be discussed as a limitation.

We thank the reviewer for this insightful comment. In accordance with your suggestion, we have revised the Discussion section to more clearly acknowledge the limitations of our outcome assessment. Specifically, we now discuss the fact that our evaluation was based primarily on histological analysis of cartilage, and we agree that including biochemical markers and assessing the synovium could have provided better insights into HGF-mediated mechanisms and the broader therapeutic effects of synovial MSCs in OA. We hope this addition addresses your concern. We have added the following sentences to the limitations:

“Finally, our in vivo outcome assessment relied primarily on histological evaluation. Including biochemical markers of cartilage composition and comprehensive analysis of synovial tissue responses could provide additional mechanistic insights into HGF-mediated chondroprotection.”

- Linked to the previous point, it should be emphasized that this is an observational study but not a mechanistic one. This study reveals that HGF plays some role on the outcome of MSC therapy in OA, but does not provide information about which is this role. Thus, any claim about mechanisms should be done very carefully. In addition, regarding the in vitro part of the study, only chondrogenesis is assessed, however (as authors state in the discussion), MSC therapeutic effects in OA are mainly mediated via paracrine signaling and not by direct differentiation, so in vitro assays could have also been designed more in that direction. I consider that this should at least be discussed as a limitation.

We thank the reviewer for this valuable and constructive comment. In response, we have revised the Discussion section to include an explicit statement acknowledging that this is an observational study, not a mechanistic one. We agree that while our results support a role for HGF in MSC-mediated cartilage protection, the specific signaling pathways remain to be elucidated. We have also noted that the in vitro analysis focused solely on chondrogenesis, and that additional assays addressing paracrine signaling (such as cytokine profiling or anti-inflammatory assays) would have provided complementary insights given the known mechanism of MSC action in OA. These points have now been incorporated as limitations in the revised manuscript, as follows:

“Our mechanistic analysis focused specifically on HGF function through siRNA knockdown. While this approach demonstrated the contribution of HGF to MSC efficacy, we did not conduct a comprehensive evaluation of whether *Hgf* knockdown affected other chondroprotective factors, such as PRG4 or BMP2/6 [3], nor did we perform rescue experiments to isolate the specific contribution made by HGF. Future studies incorporating secretome analysis and rescue experiments would provide more definitive mechanistic insights.”

Specific comments:

Introduction:

- In addition to what has been commented under general comments, I would recommend including in the introduction a brief explanation about the rational for choosing these two strains of rats (ACI and Lewis) to establish a MHC-mismatched transplantation scenario.

- Goals at the end of the introduction section should be adjusted as suggested under general comments, especially regarding the MHC-mismatching not being a goal itself but a study condition. Focus should be placed on the role of HGF in MSC therapeutics from an observational perspective.

As already mentioned, we have comprehensively rewritten the Introduction to address these comments.

Material and methods:

Animals:

- Any particular reason to use all male donors and all female recipients? Using half males and half females as recipients could have allowed to also accounting for the sex effect.

We have added the following paragraph to the Discussion:

“The present analysis did not include sex-based cell tracking, although male donor rats and female recipient rats have been used in the past to enable the potential tracking of donor cells using Y chromosome-specific markers [29]; However, our aim was not to evaluate sex-specific effects, and we believe the sex difference did not influence the therapeutic outcomes observed.”

Isolation of rat synovial MSCs

- Under the previous section “Animals”, in lines 86-87, it is stated that "To establish MHC-mismatched synovial MSCs, 30 male ACI rats (...) were purchased", however here it is mentioned that 15 rats were used to create a pool of synovial MSCs. What the other 15 ACI male rats were used for?

We used the other 15 ACI animals to confirm the synovial tissue collection procedure. We have added the following sentence:

“To establish MHC-mismatched synovial MSCs, 30 four-week-old male ACI rats (ACI/N Slc), weighing 50–70 g, were purchased from Japan SLC, Inc. (Hamamatsu, Japan). Fifteen of these rats were used for pooled synovial MSC preparation for transplantation, and the remaining 15 were used to confirm the tissue harvesting protocol.”

- If I properly understood, all in vitro and in vivo assays were conducted with passage 0 and passage 1 cells. Were these passages selected for any particular reason? Early passages such as 0 and 1 may present higher cell heterogeneity compared to later passages, which are more frequently used for therapeutic purposes to provide a more homogeneous cell population.

We have added the following paragraph to the Discussion:

“Early passage synovial MSCs (passages 0 or 1) were selected for both in vitro and in vivo experiments. This decision was based on previous studies by Okuno et al. [6] and Ozeki et al. [3], which also employed early passage cells. Clinical applications of synovial MSCs typically involve cells at passage 0 or 1 [4, 23-25], since extended passaging can reduce MSC stemness and regenerative capacity [12].”

- Were synovial MSCs characterised by any means? i.e immunophenotype, tri-lineage differentiation?

Rat synovial MSCs have been previously characterized as mesenchymal stem cells based on their trilineage differentiation potential and chondrogenic capacity [6]. The cells used in this study were established using the same protocol; therefore, only their chondrogenic differentiation potential was evaluated. A citation to Okuno et al. [6] has been added at the end of the “Isolation of rat synovial MSCs” section in the Methods to support the established protocol and prior characterization of these cells.

Analysis of Hgf expression

- At what time-points was HGF gene expression and protein secretion assessed? For gene expression it is stated "in the cryopreserved stock". Does it mean in the cells frozen after 24h of transfection? For protein secretion (ELISA), time points are not stated but in Results (figure 1) one can see day 1, 3 and 7. Please ensure that experiment design is explained with enough detail under methodology to match with what is presented under results.

- Since the synovial MSCs used consisted of a pool from 15 donors, I guess only this one biological (pooled) replicate was used, and that what is represented in figures are technical replicates. For example, for ELISA results (fig 1.C) three replicates are represented, but for gene expression it is not clear. Please explain these methodological details about biological and technical replicates under methodology section.

- Line 137: analysis method for gene expression should be 2^-ΔΔCt instead of 2^-ΔCt.

- Please briefly explain how the HGF ELISA was performed. Even if it was done according to kit's manufacturer's instructions, a brief explanation should be provided including any procedure on the supernatants (centrifugation, filtration, freezing...), any dilution of the samples, points in the standard curve, absorbance length and equipment used for absorbance reading, as well as negative controls used (i.e. what is represented as "w/o MSCs" in figure 1.C). This information cannot be exclusively placed in the figure captions but needs to be in the main text so the reader can get the necessary details on it.

We have rewritten the methods as follows:

“**Analysis of Hgf expression**

Total RNA was isolated from synovial MSCs transfected with either *Hgf* siRNA or negative control siRNA using the RNeasy Mini Kit (Qiagen, Venlo, Netherlands). The cells used for RNA extraction were obtained from a cryopreserved stock that had been frozen 24 h after siRNA transfection. Complementary DNA (cDNA) was synthesized using the High Capacity RNA-to-cDNA Kit (Thermo Fisher Scientific). Quantitative PCR was performed in triplicate for each sample using the TaqMan Gene Expression Master Mix (Thermo Fisher Scientific) and a CFX384 Touch Real-Time PCR Detection System (Bio-Rad Laboratories, Hercules, CA), according to the manufacturer’s instructions.

TaqMan™ gene expression assays were used to quantify rat *Hgf* (Assay ID: Rn00566673_m1) and the endogenous control *Actb* (Assay ID: Rn00667869_m1) (Thermo Fisher Scientific). Gene expression levels were calculated using the 2^–ΔΔCt method, with normalization to *Actb*. The synovial MSCs used in this study were pooled from 15 individual ACI rat donors, and all gene expression analyses were conducted using this single pooled cell population, representing one biological replicate. Quantitative PCR was performed in technical triplicates, and the values presented in the figures reflect these technical replicates.

**Analysis of HGF protein secretion**

HGF protein secretion was assessed using a Rat HGF ELISA Kit (Abnova, Taipei, Taiwan) in synovial MSCs transfected with either *Hgf* siRNA or negative control siRNA. The cryopreserved cells were thawed, seeded, and cultured in cell isolation medium for up to 7 days. Culture supernatants were collected on days 1, 3, and 7. Prior to analysis, the supernatants were centrifuged at 300 × *g* for 5 min to remove cell debris and stored at –80°C. Undiluted supernatants were used for ELISA, and culture medium without MSCs served as a negative control.

The ELISA procedure was performed according to the manufacturer’s protocol. Briefly, 100 µL of standards or samples was added to each microplate well and incubated for 2 h at room temperature. A 100 µL volume of biotinylated detection antibody was then added to each well, followed by a 2 h incubation at room temperature (RT). After washing, 100 µL of streptavidin–peroxidase conjugate was added and incubated for 40 min at room temperature. Following another wash, 90 µL of chromogen substrate was added. After a 7 min incubation, the reaction was stopped with 100 µL of stop solution. Absorbance was measured at 450 nm using a Cytation 5 plate reader (BioTek, Agilent Technologies, Santa Clara, CA).

As with the gene expression analysis, ELISA assays were performed in technical triplicates using supernatants derived from the same pooled MSC batch (one biological replicate).”

In vitro chondrogenesis assay

- It is my understanding, according to the results (figure 2) that both HGF-silenced MSCs and control MSCs were used in chondrogeneic assays, however this is not detailed in the methodology. Please state it clearly in the methodology, the reader needs to get clear and direct information from the text and not to guess based on figures. Please also state the number of biological and technical replicates, as mentioned for gene expression/protein secretion of HGF.

We have revised the Materials and Methods section to clarify the use of both Hgf-silenced MSCs and control MSCs in the chondrogenesis assay, and to specify the number of biological and technical replicates, as follows:

**“In vitro chondrogenesis assay**

Synovial MSCs transfected with either *Hgf* siRNA or negative control siRNA were used to assess chondrogenic differentiation. Cells were derived from a single cryovial of pooled, cryopreserved MSCs, representing one biological replicate. For each group, three chondrogenic pellets were prepared as technical replicates (n = 3 per group) by suspending 2.5 × 10⁵ cells per pellet in 1 mL of chondrogenic medium.”

- Please briefly describe the immunostaining procedure, even if previously described in bibliography (primary antibody clone, secondary antibody if applies, antibody dilution, etc.).

We have revised the Materials and Methods section as follows:

“For histological evaluation, the pellets were fixed, embedded in paraffin, sectioned at 5 μm thickness, and stained with safranin O and toluidine blue. For immunohistochemistry, type II collagen was detected using a purified anti-human type II collagen IgG antibody (clone II-4C11; Kyowa Pharma Chemical Co., Ltd., Toyama, Japan) diluted 1:200 in Antibody Diluent (DAKO, Glostrup, Denmark). The secondary antibody was Histofine Simple Stain Mouse MAX-PO(R) (Nichirei Corp., Tokyo, Japan). Sections were incubated with the primary antibody for 60 min at room temperature, followed by incubation with the secondary antibody for 30 min at room temperature. Immunoreactivity was visualized using 3,3′-diaminobenzidine (DAB; Muto Pure Chemicals Co., Ltd., Tokyo, Japan) as the chromogen.”

Anterior cruciate ligament transection (ACLT) surgery and MSC injection

- In line 89 (Animals section), it is stated that 34 female Lewis rats were purchased. Based on the abstract, 20 knees were included per group. According to this section, both knees of each rat were used. One can infer that 30 rats were used, divided into three groups of 10 rats per groups, and using both knees of each rat, thus making 20 knees per group. Two comments about this:

- This information about group distribution should be clearly explained in this section with exact number of animals and knees, and not left to be inferred by the reader from different parts of the manuscript.

- If 34 rats were purchased but 30 were used for the assay, what the other 4 rats were used for? One may guess that extra animals were purchased in case animals in the study needed to be replaced, but this should not be inferred but crystal clear explained. The destination of the unused/discarded animals (guess euthanasia?) should also be disclosed.

- Please briefly explain anaesthetic and pain management protocols for this particular ACLT surgery.

- Please explain procedure for intraarticular injections in rats. Were these done under general anaesthesia? With which needle size? Volume injected per joint? Vehicle substance? Were cells administered directly after thawing or were cultured for a few days after thawing to readjust conditions? If the former, viability of cells post thawing?

We have totally rewritten this section as follows:

“**Anterior cruciate ligament transection (ACLT) surgery and MSC Injection**

Following an acclimation period of at least 7 days, bilateral anterior cruciate ligament (ACL) transection was performed in 30 Lewis rats by lateral dislocation of the patella to expose the joint, as previously described [3]. All surgeries were conducted under isoflurane anesthesia. Anesthesia was induced with 3–5% isoflurane in an induction chamber and maintained at 1–3% via a nose cone. Body temperature was maintained throughout the procedure, and the anesthesia depth and the general condition of the animals were continuously monitored. After surgery, the rats were allowed free movement in their cages. To minimize procedural pain, surgeries were performed under adequate anesthesia by an experienced operator. Postoperative monitoring was carried out daily, and no animals showed signs of severe pain or distress.

One week after surgery, the rats were randomly divided into three groups of 10 animals each (n = 10), resulting in 20 knee joints per group (n = 20). Under isoflurane anesthesia, a 28-gauge needle was used for intra-articular injection of 50 μL of the following assigned treatments into the knee joints of the animals in each group:

1. *Hgf* siRNA-transfected MSCs (1 × 10⁶ cells),
2. Negative control siRNA-transfected MSCs (1 × 10⁶ cells), or
3. Vehicle alone (no MSCs).

Each treatment was administered once weekly for 7 consecutive weeks. MSCs were thawed immediately before injection, and cell viability was confirmed to be ≥90% at the time of administration.

The sample size was based on prior in vivo studies [3] to ensure sufficient power for detecting group differences. Of the 34 animals initially purchased, 4 were excluded prior to surgery due to health concerns and were euthanized in accordance with institutional guidelines.”

Macroscopic evaluation

- Line 171: even if it might be obvious, please state that at this time point (8 weeks) animals were euthanized as explained before for tissue harvesting.

We have added the following explanation:

“At 8 weeks after ACLT surgery, all animals were euthanized by exsanguination through abdominal aortic resection under 2.5% isoflurane inhalation anesthesia administered via mask.”

Statistical analysis

- How was normality assessed, e.g. Shapiro Wilk, Kolmogorov Smirnov...? Since non-parametric tests were used (Kruskall Wallis, Mann-Whitney) I guess that normality was checked and not met. Please clarify.

We thank the reviewer for this important methodological question. We did not perform formal normality testing (such as Shapiro–Wilk or Kolmogorov–Smirnov tests) prior to the statistical analysis. Instead, we chose to use nonparametric tests (Kruskal–Wallis and Mann–Whitney U tests) a priori as our primary statistical approach. This decision was made to ensure robust analysis regardless of the underlying data distribution, particularly given our relatively small sample sizes, which can make normality testing less reliable. Nonparametric tests are distribution free and provide valid results without requiring assumptions about normal distribution, making them appropriate for our experimental design.

- In addition, when three groups are compared (e.g. control, HGF-silenced MSCs, control MSCs), the customary strategy is to run a test like Kruskall-Wallis with a posthoc such as Dunns or similar, and not to subsequently conduct pairwise analysis like Mann-Whitney. Please revise and consider amendment.

We thank the reviewer for the insightful comment. Although Dunn’s test is commonly used as a post hoc test following Kruskal–Wallis analysis, we chose to perform pairwise comparisons using the Mann–Whitney U test with Bonferroni correction for multiple comparisons. This approach is also widely accepted and provides appropriate control of type I error. We have now revised the Methods section to explicitly state that Bonferroni correction was applied for all pairwise analyses following Kruskal–Wallis testing. We believe that this approach ensures both statistical rigor and interpretability of the results.

- Why customary p<0.05, p<0.01 and p<0.001 were not used but instead arbitrary p value levels are considered? It is not technically incorrect, but a bit odd to make this choice.

Thank you for your comment. We used adjusted significance thresholds (e.g., p < 0.0167, p < 0.0033, p < 0.00033) due to the application of Bonferroni correction for multiple pairwise comparisons. These thresholds were calculated by dividing the conventional alpha levels by the number of comparisons to control the family-wise error rate. This is why the commonly used thresholds of p < 0.05, p < 0.01, and p < 0.001 were not used directly.

In response to the comments mentioned above, we have thoroughly revised the Statistical Analysis section as follows:

“Statistical analysis

Statistical analyses were performed using GraphPad Prism version 5.04 (GraphPad Software Inc., San Diego, CA, USA). Nonparametric methods were used throughout this study to avoid assumptions about data distribution and to ensure robustness, particularly given the small sample sizes.

Group differences were first assessed using the Kruskal–Wallis test. When significant results were obtained, pairwise comparisons were performed using Mann–Whitney U tests with Bonferroni correction for multiple comparisons. The Bonferroni method was selected to control the family-wise error rate. Accordingly, the significance threshold was adjusted to p < 0.0167 (0.05 divided by 3 comparisons).

Statistical significance in the results was indicated as follows: p < 0.0167, p < 0.0033, p < 0.00033, depending on the number of comparisons made. All data are presented as medians with interquartile ranges. A p-value < 0.0167 was considered statistically significant.”

Results

Hgf knockdown

- Figure 1a does not provide much information. It could be removed or, if kept, it should be enriched with further details like a whole study design representation of the in vitro assays (gene expression, protein secretion, chondrogenesis), the n of animals, etc.

In response to Reviewer #2’s comment, we have removed Figure 1a from the manuscript, as it provided limited information.

- Figure 1b: the amplification plot is not needed and just the table should be kept. As mentioned in methodology, number of biological and technical replicates should be included. I guess it was only 1 replicate and thus there is no mean or deviation to show in terms of gene expression. In the table, it would be useful to include value 1 for the reference sample, even to represent these numbers (1 and 0.3) in a bar graph for visually representing the downregulation of HGF.

In response to Reviewer #2’s comments, we have removed the amplification plot from Figure 1b, as suggested. We have also added the value “1” for the reference sample (Cont si) in the table to clarify the relative expression level.

- Figure 1c: Were statistically significant differences found among negative control (whatever it is, w/o cells), control MSCs and HGF-silenced MSCs in terms of HGF secretion? Or along the time i.e. among 1, 3 and 7 days? This should also be explained in the main text.

Thank you for your comment. Although we performed statistical testing, the sample size (n = 3 per group) was considered insufficient to draw definitive conclusions about statistical significance. Therefore, we chose not to include p-values in the main text. However, visual inspection of the data suggests that HGF secretion was markedly suppressed in the Hgf siRNA-treated MSC group compared to the control siRNA-treated group at all time points and remained at levels comparable to the negative control (w/o MSCs).

We have revised the Results section as follows:
“While synovial MSCs treated with control siRNA showed an increase in HGF protein levels on days 3 and 7, MSCs treated with *Hgf* siRNA showed levels similar to the medium-only controls (negative control), indicating suppression of HGF secretion through day 7 (Fig. 1b).”

In vitro analysis

- Were statistically significant differences found between control and silenced-HGF MSCs in terms of weight or diameter of the chondrogeneic pellets produced?

Thank you for your comment. We did not perform statistical analysis for the weight or diameter of the chondrogenic pellets because the sample size (n = 3 per group) was considered too small to yield statistically reliable results. Instead, we evaluated the trend descriptively and observed that the pellets derived from Hgf-silenced MSCs tended to be smaller and lighter compared to those derived from control MSCs. However, we acknowledge that statistical analysis with a larger sample size would be necessary to confirm the significance of this observation.

- Number of biological and technical replicates in the chondrogeneic assay?

The chondrogenic assay was conducted using three technical replicates (n = 3) for each MSC group. These replicates were derived from a single pooled MSC sample; thus, they represented one biological replicate, as already mentioned.

- Figure 2: Even if quantification of histological parameters in chondrogeneic pellets has not been performed, there are some differences between control and silenced HGF MSCs that can be qualitatively described in the main text, such as different staining intensity of Safranin O, for example.

We have added the following qualitative description of the cartilage matrix staining to the Results section:

“Histological analysis showed that both groups produced cartilage matrix that stained positively with toluidine blue, safranin O, and type II collagen (detected by immunohistochemistry using DAB as the chromogen). However, the staining intensity of safranin O appeared weaker in the *Hgf* siRNA-treated group, and the area of matrix staining relative to the overall pellet was slightly reduced. These findings suggest that HGF secreted by synovial MSCs may influence the matrix production capacity during chondrogenic differentiation.”

- Figure 2 caption needs to provide more detail into all the data presented (types of staining and immunostaining).

We have rewritten the legends in Figure 2 as follows:

**“Fig 2. Effect of HGF suppression on chondrogenic differentiation of synovial MSCs in vitro.**

Macroscopic and histological images showing cartilage pellet morphology after chondrogenic differentiation of synovial MSCs treated with control siRNA (Cont si) or Hgf siRNA (HGF si). Histological staining included hematoxylin and eosin (HE), Toluidine blue, and Safranin O. Type II collagen was detected by immunohistochemistry using DAB as the chromogen. Graphs indicate the quantification of pellet weight (mg) and length (mm). Each point represents a single pellet (n = 3).”

In response to the comments, we have rewritten the results as follows:

“**In vitro analysis**

Macroscopic observation of cartilage pellets after 3 weeks of differentiation (n = 3 per group) revealed that *Hgf* siRNA-treated pellets tended to be smaller in both weight and diameter compared to control siRNA-treated pellets (Fig. 2). Statistical analysis was not performed due to the small sample size and the fact that pellets were derived from a single pooled MSC batch (biological replicate = 1). These properties were considered insufficient to yield reliable statistical conclusions.

Histological analysis showed that both groups produced cartilage matrix that stained positively with toluidine blue, safranin O, and type II collagen (detected by immunohistochemistry using DAB as the chromogen). However, the staining intensity of safranin O appeared weaker in the *Hgf* siRNA-treated group, and the area of matrix staining relative to the overall pellet was slightly reduced. These findings suggest that HGF secreted by synovial MSCs may influence the matrix production capacity during chondrogenic differentiation.”

Discussion

- Lines 414 – 416: “The significant reduction in MSC therapeutic efficacy following Hgf knockdown suggests that this growth factor plays a crucial role in orchestrating the complex interplay of these protective mechanisms”. This is an overstatement. First, even though the score reduction was statistically significant over control, such reduction was not so dramatic. Second, and more importantly, from these results it cannot be concluded which exact mechanisms plays HGF in relation to the multimodal action of MSCs, since other mediators and their interactions have not been assessed (please also see general comments).

We have rewritten the paragraphs as follows:

“The chondroprotective effects of synovial MSC injections in OA are mediated through complex paracrine mechanisms rather than via direct cellular differentiation. Following intra-articular injection, MSCs primarily localize to the synovium, where they maintain their stem cell properties [3] and orchestrate therapeutic actions by secreting multiple bioactive factors. These secreted mediators include HGF, TSG-6 (which exerts anti-inflammatory effects [19]), PRG-4 (lubricin; which enhances joint lubrication and cartilage homeostasis [20]), and BMP-2/6 (which promotes chondrocyte differentiation and matrix synthesis [21]). Together, these factors create a comprehensive therapeutic milieu that simultaneously addresses the key pathological features of OA: inflammation, lubrication deficiency, and cartilage degeneration [22].

In the present study, *Hgf* knockdown in synovial MSCs resulted in a moderate but statistically significant reduction in both in vitro chondrogenic capacity and in vivo therapeutic efficacy, demonstrating that HGF contributes meaningfully to the overall chondroprotective effects of MSCs. However, since other key paracrine mediators were not evaluated in this study, the relative contribution of HGF within the broader multimodal therapeutic framework remains to be fully elucidated. Future studies examining the individual and synergistic roles of various secreted factors will be necessary to obtain a comprehensive understanding of the mechanistic hierarchy underlying MSC-mediated cartilage protection.”

- Lines 420 – 424: The choice of the mismatched scenario using two rat strains should be explained in the introduction or in the study design section so the reader can understand the suitability of the mismatched design from the beginning of the manuscript.

*- Lines 420 – 424:*

*“Our major mismatched model involved the transplantation of ACI rat cells into Lewis rats. The major histocompatibility complex (MHC) class I genes in rats consist of A, Pa, F, B, D, and E loci, and because the ACI and Lewis rats differ in all their class I major antigens except for the F locus, this transplantation represented a major mismatch [5, 25].”*

We have incorporated this information into the Introduction section to ensure that the rationale for using the mismatched transplantation model was clear right from the beginning of the manuscript.

- Lines 424 – 425: This sentence should be revised. As already mentioned, the lack of MHC-matched control (i.e. syngenic) does not allow inferring if the cells would have promoted more or less effect depending on the matching degree. Maybe matched cells would have not elicited more therapeutic effects in the conditions of this study, so it cannot be inferred that it is a positive results that mismatched cells still elicited therapeutic effect.

*- Lines 424 – 425:*

*“Despite these severe conditions, which were likely to lead to immune rejection, therapeutic effects were still achieved.”*

Editor’s notes: The responses to the reviewers are very well written and clear. The authors convincingly show that they understood the concerns raised by the reviewers and have provided polite and concise responses.

We have entirely deleted the paragraph related to the comments because this content has been moved to the introduction.
