# Supplementary material for: MHC-mismatched synovial mesenchymal stem cell injections delay knee osteoarthritis progression through hepatocyte growth factor secretion in rats
Source: PLoS One. 2025 Oct 3;20(10):e0333117. doi: 10.1371/journal.pone.0333117 (PMC12494247; doi:10.1371/journal.pone.0333117)
Supplement: Attachment — Submitted filename: Kitahashi Allo Plos Rebuttal wo cover 250904 1720.docx [file pone.0333117.s004.docx]

Review Comments to the Author

Reviewer #2:

Authors have done a remarkable job revising the manuscript, which has significantly improved. It is particularly worth of mentioning the great work with the Introduction, which is convincing yet concise and clearly highlighting the main points. I consider that the manuscript is in good shape for acceptance, yet a few minor points need attention:

- Regarding the procedure for MSC injection, the vehicle substance is not yet disclosed. May it be CP-1 High Grade cryoprotectant used for freezing the cells? If so, please clearly state.

We appreciate the reviewer’s comment and have revised the Methods section to explicitly state the use of CP-1 High Grade cryoprotectant as the vehicle for MSC cryopreservation and injection. The relevant passages now read as follows:

“Isolation of rat synovial MSC

Rat synovial MSCs were isolated as previously described [10]. After an adaptation period of at least 7 days, male ACI rats were euthanized by exsanguination from the inferior vena cava under isoflurane anesthesia. Synovial tissue harvested from the infrapatellar fat pad of both knees from 15 rats was pooled and minced, followed by digestion with Liberase MNP-S (Roche Diagnostics Corp., IN, USA) in a water bath at 37°C for 2 h. The resulting isolated cells were cultured in α-minimum essential medium (αMEM; FUJIFILM Wako Pure Chemical Corp., Osaka, Japan) supplemented with 20% fetal bovine serum (FBS; Thermo Fisher Scientific, MA, USA) for 8 days at 37°C in 5% CO2. The cells were then harvested and cryopreserved as original stocks in CP-1 High Grade cryoprotectant (Kyokuto Pharmaceutical Industrial Co. Ltd., Tokyo, Japan) at ‑150°C (CLN-1700CWE, Nihon Freezer, Tokyo, Japan) (Passage 0). These isolation procedures were performed in three separate sessions. For experiments, synovial MSCs from the original stocks (Passage 0) were cultured for 7 days, harvested, and cryopreserved in CP-1 High Grade cryoprotectant (Passage 1). The cells were used for subsequent analyses without any sorting [6]. For cryopreservation, CP-1 High Grade was supplemented with 25% human recombinant albumin expressed in plants (Albumin, Human, recombinant expressed in plants; FUJIFILM Wako Pure Chemical Corp.), resulting in a final composition of 6% hydroxyethyl starch (HES), 5% dimethyl sulfoxide (DMSO), and 4% human recombinant albumin.”

“One week after surgery, the rats were randomly divided into three groups of 10 animals each (n = 10), resulting in 20 knee joints per group (n = 20). Under isoflurane anesthesia, a 28-gauge needle was used for intra-articular injection of 50 μL of the following assigned treatments into the knee joints of the animals in each group:

1. *Hgf* siRNA-transfected MSCs (1 × 10⁶ cells),
2. Negative control siRNA-transfected MSCs (1 × 10⁶ cells), or
3. Vehicle alone (CP-1 High Grade cryoprotectant without MSCs).”

- Regarding the sex of the animals of the study: I understand that it is a common approach to use males as donors and females as recipients in order to track the cells using Y chromosome markers, and I think this can be a valid point when designing the study, even though tracking of male donor cells was not pursued. I also understand that including a gender dimension in the in vivo studies can be challenging and require extra resources. However, I feel that the statement “we believe the sex difference did not influence the therapeutic outcomes observed” (lines 559-560 of the Discussion) is not supported by evidence (e.g. doi: 10.21037/atm-23-1546) and thus should be removed.

We acknowledge the reviewer’s important observation regarding our statement on sex differences in therapeutic outcomes. As the reviewer correctly points out, our assertion that "we believe the sex difference did not influence the therapeutic outcomes observed" (lines 559–560) was not supported by direct evidence, since our study was not designed to systematically investigate sex-based differences in treatment response. Recognizing the growing body of literature emphasizing the influence of sex on therapeutic outcomes, and given that we did not collect data to address this question, we agree with the reviewer's concern and have therefore removed this unsupported statement from the manuscript. We have rewritten the paragraph as follows:

“The present analysis did not include sex-based cell tracking, although male donor rats and female recipient rats have been used in the past to enable the potential tracking of donor cells using Y chromosome-specific markers [29]; However, our aim was not to evaluate sex-specific effects, and this limitation should be considered when interpreting the results.”

- In line 480 of the Discussion, the statement “even in the presence of strong allogeneic immune responses” is an assumption, since the immune response against the MSCs was not evaluated. Please consider removing this sentence to avoid misleading information.

We are grateful to the reviewer for highlighting this important issue. The reviewer is correct that our statement “even in the presence of strong allogeneic immune responses” (line 480) was an assumption rather than a conclusion supported by data, as our study design did not directly evaluate immune responses against the transplanted MSCs. Recognizing that such assumptions without proper validation may mislead readers, we agree with the reviewer’s suggestion and have removed this statement from the Discussion to maintain accuracy and scientific rigor.
